# Enzymolytic soybean meal improves growth performance, economic efficiency and organ development associated with cecal fermentation and microbiota in broilers offered low crude protein diets

**DOI:** 10.3389/fvets.2023.1293314

**Published:** 2023-11-17

**Authors:** Xin Zhu, Kai Gao, Yongqiang Qi, Guiqin Yang, Haiying Liu

**Affiliations:** College of Animal Science and Veterinary Medicine, Shenyang Agricultural University, Shenyang, China

**Keywords:** broiler, low protein, soybean meal, growth performance, microbiota, economic efficiency

## Abstract

The objective of this experiment was to determine the effect of low crude protein (CP) diets containing increasing amounts of enzymolytic soybean meal (ESBM) on growth performance, economic benefit and organ development and the role of cecal fermentation and microbiota in broilers. A total of 360 one-day-old Arbor Acres chicks were randomly allocated into 6 groups with 6 replicates and 10 chicks each. The six dietary treatments consisted of a standard high-CP diet (PC), a low-CP diet (NC), and an NC diet with 0.5, 1.0, 1.5%, or 2.0% ESBM. The experiment lasted for 42 days. Compared to PC, NC showed decreased (*p* < 0.05) average daily gain (ADG) in broilers from 22 to 42 days and from 1 to 42 days, while increasing levels of ESBM quadratically increased (*p* < 0.05) ADG from 1 to 42 days. Feed cost and total revenue in the NC were lower (*p* < 0.05) than that in the PC, while supplementation with ESBM in the NC linearly increased (*p* < 0.05) net profit and economic efficiency in broilers. There were significant differences (*p* < 0.05) in the liver, proventriculus and gizzard indices between the PC and NC groups, and supplementation with ESBM linearly increased (*p* < 0.05) the relative weights of liver, pancreas, proventriculus and gizzard in broilers at 42 days of age. The PC group had a higher cecal acetic acid concentration at 21 days and propionic acid concentration at both 21 and 42 days than the NC group (*p* < 0.05). Cecal acetic acid and propionic acid concentrations linearly increased (*p* < 0.05) with increasing levels of ESBM in broilers at 42 days of age. No significant differences in ACE, Chao1, Shannon and Simpson indices were observed among groups (*p* > 0.05), while the cecal abundances of *Bacteroides*, *Faecalibacterium* and *Clostridium IV* increased (*p* < 0.05) with the increasing level of ESBM in the low-CP diets. In conclusion, feeding ESBM improved economic efficiency, digestive organ development, cecal fermentation and microbial community composition, and up to 2.0% ESBM addition had no negative effect on the growth performance in broilers fed low CP diets.

## 1 Introduction

The world population is estimated to increase from the current 8 billion to 9.6 billion by 2050, leading to a very increasingly urgent demand for animal protein (1). On account of its large scale and intensification with high yields and low price, broiler chicken feeding has become one of the most growing food animal production sectors worldwide (2). As the feed cost covers up to 70% of the total production cost in commercial broiler production, the request for lowering diet cost and enhancing feedstuff utilization efficiency is always strong (3). Among feed ingredients, vegetable proteins and their processed byproducts, such as soybean meal (SBM), are mostly included in broiler diets. However, the shortage and high price of these protein feedstuffs often have a negative impact on the profit of the broiler sector, especially in protein feedstuff-less self-sufficient countries and regions of the world, such as China and the European Union (4). The adoption of reduced levels of crude protein (CP) in diets and increased feed efficiency is, therefore, contributing to sustainable chicken-meat production (5).

In recent years, from the perspective of nutrition, a strategy of reducing the inclusion of CP in diets to improve the sustainability of broiler production has been widely evaluated (6–8). Along with crystalline free amino acids being commercially available at manageable levels of price, reducing dietary CP content and supplementing free amino acids to meet ideal amino acid profiles can allow for refinement of diet formulation. The benefits of feeding a low-CP diet to broilers are not only decreasing the excretion of nitrogen originating from dietary protein responsible for environmental protection but also reducing the cost of animal feeding, contributing to broiler industry development (6). In terms of growth performance, an intriguing conundrum still exists in broilers fed low-CP diets. It was well documented that reducing dietary CP content by <2% and simultaneously supplementing crystalline amino acids, including indispensable and dispensable amino acids, did not compromise growth performance in broilers (8, 9). Nevertheless, some researchers also found negative effects of low-CP diets on growth performance in broilers when the CP level was reduced by 2% (10). A recent meta-analysis study performed by Alfonso-Avila et al. (6) found that reducing dietary CP levels was generally completed by replacing soybean meal with corn, and the average daily gain and feed conversion ratio deteriorated in broilers fed diets when the CP level decreased by 2%. Furthermore, by reducing the dietary CP level by 4% or more, the growth performance of broilers is inevitably depressed even when all feed-grade amino acids are supplied. To meet the nutrient requirements and improve feed efficiency, it may be crucial that the extent of CP reduction is not exceeded by 2% in standard high-CP diets to maintain satisfactory performance of broilers (11).

In current broiler diet formulation, SBM is the most widely used vegetable protein source with balanced amino acid profiles. However, there are also many antinutritional factors, including trypsin inhibitors, antigenic proteins, and phytic acids which can hamper the digestion and absorption of nutrients, thus leading to a negative influence on the health and growth performance of broilers (12). To overcome this disadvantage, various methods of processing, including extraction, heating, and fermentation, are used to degrade macromolecular proteins and eliminate these antinutritional factors (13). The fermentation of SBM is cost-effective, but the quality of fermentation related to reducing the antinutritional factors in SBM can be mainly attributed to the fermentation process, which includes temperature, duration, pH, and microorganisms used in the process (14). Compared with those methods, enzymatic hydrolysis is a safe, time-saving technological process that is widely accepted to disrupt peptide or disulfide bonds, improve protein or peptide solubility and facilitate intestinal absorption (15). It has been reported that the inclusion of enzymolytic soybean meal (ESBM) in the diet to replace some other protein sources, such as SBM, fermented SBM or fish meal, can improve growth performance, immune function and intestinal health (16). However, to the best of our knowledge, there is a shortage of studies concerning the use of ESBM in reduced-CP diets. Hence, the objective of this study was to investigate the effects of ESBM supplementation on growth performance, economic efficiency, organ development, cecal fermentation and the microbial community in broilers fed low-CP diets.

## 2 Materials and methods

### 2.1 Animals, diets, and experimental design

The ESBM was provided by K&P Ltd. (Shenyang, China), and the nutrient level and amino acid composition of ESBM are shown in Supplementary Table S1.

A total of 360 one-day-old Arbor Acres broiler chicks with similar initial live weights from a local commercial hatchery (Shenyang, China) were randomly allocated into 6 groups with 6 replicates and 10 chicks (half female and half male) per replicate. Six dietary treatments were as follows: a standard high-CP diet (positive control, PC), a low-CP diet (negative control, NC), and an NC diet +0.5, 1.0, 1.5 or 2.0% ESBM. A PC diet was formulated at 215 g/kg and 195 g/kg CP for the starter (1–21 days) and grower (22–42 days) periods, respectively, to meet CP requirements in current Chinese broiler feeding sectors (NY/T33-2004, Ministry of Agriculture of the People’s Republic of China, 2004). An NC diet was created from a PC diet by reducing 10 g/kg CP but supplementing lysine, methionine and threonine to match the amino acid profile of a PC diet. SBM of an NC diet was then replaced with different levels of ESBM to produce experimental diets (Tables 1, 2). All diets were provided as mash from 1 to 42 days.

**Table 1:** The ingredient composition and nutrient levels of diets in the starter period (1–21 days, air-dried basis, g/kg).

Ingredients	Groups					
	PC	NC	NC + 0.5%ESBM	NC + 1.0%ESBM	NC + 1.5%ESBM	NC + 2.0%ESBM
Corn	514	537	537	537	537	537
SBM	295	279	274	269	264	259
ESBM	0	0	5	10	15	20
Rice bran	50	50	50	50	50	50
DDGS	40	40	40	40	40	40
Fish meal	35	25	25	25	25	25
Extruded soybean powder	32	32	32	32	32	32
Soybean oil	10	10	10	10	10	10
Limestone	10	11	11	11	11	11
Calcium hydrophosphate	1.7	1.7	1.7	1.7	1.7	1.7
Choline chloride	0.5	1.1	1.1	1.1	1.1	1.1
Sodium chloride	1.0	1.0	1.0	1.0	1.0	1.0
Premix^1^	1.5	1.5	1.5	1.5	1.5	1.5
Lysine (98.5%)	3.4	4.2	4.2	4.2	4.2	4.2
Methionine (99%)	3.3	3.5	3.5	3.5	3.5	3.5
Threonine (98.5%)	2.6	3.0	3.0	3.0	3.0	3.0
Total	1,000	1,000	1,000	1,000	1,000	1,000
**Nutrient levels** ^**2**^						
Metabolizable energy (MJ/kg)	12.78	12.80	12.80	12.80	12.80	12.80
Crude protein	215	205	205	206	205	206
Ether extract	70	70	69	69	69	69
Calcium	8.9	8.9	9.0	9.1	9.1	9.2
Total phosphorus	5.2	4.9	4.9	4.9	4.9	4.8
Available phosphorus	3.8	3.9	3.9	3.9	3.9	3.7
Lysine	14.8	14.8	14.7	14.7	14.7	14.7
Methionine	6.8	6.8	6.8	6.8	6.8	6.8
Threonine	10.7	10.5	10.5	10.5	10.5	10.5

^1^The premix provided the following per kg of the diets: vitamin A, 10,000 IU; vitamin D_3_, 1,000 IU; vitamin E, 25 IU; vitamin K, 0.5 mg; vitamin B_1_, 2 mg; vitamin B_2_, 8 mg; vitamin B_6_, 4.5 mg; vitamin B_12_, 0.01 mg; biotin, 0.25 mg; folic acid, 0.5 mg; nicotinamide, 40 mg; Fe, 100 mg; Cu, 25 mg; Mn, 120 mg; Zn, 80 mg; I, 10 mg; Se, 0.15 mg. ^2^All were calculated values.

**Table 2:** The ingredient composition and nutrient levels of diets in the grower period (22–42 d, air-dried basis, g/kg).

	Groups					
Ingredients	PC	NC	NC + 0.5%ESBM	NC + 1.0%ESBM	NC + 1.5%ESBM	NC + 2.0%ESBM
Corn	550.5	569	569	569	569	569
SBM	98	74	69	64	59	54
ESBM	0	0	5	10	15	20
Rice bran	68	70	70	70	70	70
DDGS	48	50	50	50	50	50
Fish meal	29	25	25	25	25	25
Extruded soybean powder	158	163	163	163	163	163
Soybean oil	19	18	18	18	18	18
Limestone	8	8	8	8	8	8
Calcium hydrophosphate	10	10	10	10	10	10
Choline chloride	0.5	0.5	0.5	0.5	0.5	0.5
Sodium chloride	2.5	2.5	2.5	2.5	2.5	2.5
Premix^1^	1.5	1.5	1.5	1.5	1.5	1.5
Lysine (98.5%)	2.5	3.3	3.3	3.3	3.3	3.3
Methionine (99%)	2.7	2.9	2.9	2.9	2.9	2.9
Threonine (98.5%)	1.8	2.3	2.3	2.3	2.3	2.3
Total	1,000	1,000	1,000	1,000	1,000	1,000
**Nutrient levels** ^**2**^						
Metabolizable energy (MJ/kg)	13.38	13.39	13.39	13.40	13.40	13.40
Crude protein	195	185	185	185	185	185
Ether extract	90	88	87	87	87	87
Calcium	8.7	8.6	8.7	8.8	8.9	8.9
Total phosphorus	5.3	5.2	5.1	5.1	5.1	5.1
Available phosphorus	3.8	3.8	3.8	3.8	3.8	3.8
Lysine	12.7	12.6	12.7	12.7	12.6	12.6
Methionine	6.0	6.1	6.1	6.1	6.1	6.1
Threonine	9.2	9.2	9.2	9.2	9.2	9.2

^1^The premix provided the following per kg of the diets: vitamin A, 10,000 IU; vitamin D_3_, 1,000 IU; vitamin E, 25 IU; vitamin K, 0.5 mg; vitamin B_1_, 2 mg; vitamin B_2_, 8 mg; vitamin B_6_, 4.5 mg; vitamin B_12_, 0.01 mg; biotin, 0.25 mg; folic acid, 0.5 mg; nicotinamide, 40 mg; Fe, 100 mg; Cu, 25 mg; Mn, 120 mg; Zn, 80 mg; I, 10 mg; Se, 0.15 mg. ^2^All were calculated values.

All broiler chicks were reared in wire-floored cages (length 120 cm × width 60 cm × height 30 cm) and were allowed free access to feed and water during the entire experiment. The ambient temperature gradually declined from 35°C at Day 1 at a margin of 0.5°C each day to 21°C, which was maintained until the end of the experiment. Light was on continuously for the whole experimental period. The broiler chicks were vaccinated against Newcastle disease and infectious bursal disease on Day 7 and Day 14, respectively. The experiment was finished at Day 42. All experimental procedures were approved by the Animal Care and Use Committee of Shenyang Agricultural University.

### 2.2 Growth performance and economic evaluation

Body weight and diet consumption were recorded weekly to monitor performance. Average daily feed intake (ADFI), average daily gain (ADG) and feed conversion ratio (FCR) were calculated as follows: ADFI = total feed intake/experimental days; ADG = (final body weight – initial body weight)/experimental days; FCR = ADFI/ADG.

An economic analysis of experimental feed and broiler production was calculated based on the prevailing market price of feed and the body weight of the broilers at the end time of the experiment, as is presented below: feed cost was calculated by multiplying cumulative feed intake throughout the feeding period by dominant prices; because a corn-soybean meal diet is widely used and feed cost accounts for approximately 70–80% of the total cost of raising broilers (17), a coefficient of 75% was used and a fixed cost of all groups was calculated: fixed cost = feed cost of PC (a corn-SBM diet in this study)/0.75 – feed cost of PC; total cost (TC) = feed cost + fixed cost; total revenue (TR) = live body weight × price/kg; net profit (NP) = TR – TC; Economic efficiency (EE) = NP/feed cost (18).

### 2.3 Sample collection

At 21 and 42 days of age, one broiler chick close to the average weight from each replicate was randomly selected and weighed after starvation for 12 h. These broiler chicks were then euthanized by cervical dislocation, and the gizzard, proventriculus, liver and pancreas were collected and weighted to calculate an organ index as follows: organ index = organ weight (g)/live body weight (kg). Both ceca from each broiler chick were taken out, and then the cecal contents were collected in a tube and stored at −80°C until further analysis.

### 2.4 Cecal volatile fatty acids

One gram of cecal contents from each replicate (*n* = 6) at 21 and 42 days of age was collected and transferred to 9 mL of distilled water, and the supernatant was extracted after centrifugation to mix with 25% metaphosphoric acid. Following filtering through a 0.22 μm filter membrane, 1 μL of sample volume was injected into a gas chromatograph system (7890B, Agilent, Wilmington, DE) equipped with a chromatographic column (30 m × 0.25 mm × 0.25 μm, FFAP, Dikma) to determine the concentrations of acetic acid, propionic acid and butyric acid.

### 2.5 Cecal microbiota and bioinformatic analysis

Total genomic DNA from cecal contents (*n* = 6) was isolated using a commercial kit (Qiagen, Beijing, China) according to the manufacturer’s instructions. The purity and concentration of the DNA were measured through 1.5% agarose gel electrophoresis and a Nanodrop spectrophotometer (Nyxor Biotech, Paris, France). Two rounds of PCR amplification were performed: In the first round, the primers (F: 5’-CCCTACACGACGTCTTCCGATCTC-3′; R: 5’-GACTFFAFTTCCTTGGCACCCGAGAATTCCA-3′) were used to amplify the variable V4 region of the bacterial 16S rRNA gene in a 30 μL reaction mixture, and the thermal cycling condition was initial denaturation at 94°C for 3 min, followed by 5 cycles at 94°C for 30 s, 45°C for 20 s and 65°C for 30 s, 20 cycles at 94°C for 20 s, 55°C for 20 s and 72°C for 30 s, and an extension at 72°C for 5 min; PCR amplification products were then purified and quantified. In the second round, a barcode sequence was introduced into the primer as follows: F: 5’-CCCTACGACGCTCTTCCGATCTGCCTACGGGNGGCWGGCWGCAG-3′; R: 5’-GACTGGAGTTCCTTGGCACCCGAGAATTCCAGACTACHVGGGTATCTAATCC-3′. The total reaction mixture was 30 μL, and the thermal cycling condition was initial denaturation at 95°C for 3 min, followed by 5 cycles at 94°C for 20 s, 55°C for 20 s and 72°C for 30 s, and an extension at 72°C for 5 min. The PCR amplified products were gel purified and then sequenced on the Illumina MiSeq platform (Sangon Biotech, Shanghai, China).

After sequencing, raw sequences were carefully trimmed, filtered and classified to obtain representative operational taxonomic units (OTUs) with above 97% similarity using the Ribosomal Database Project classifier. The microbial diversity, such as richness estimators (ACE and Chao1) and diversity indices (Shannon and Simpson), was calculated in QIIME.

### 2.6 Statistical analysis

The SPSS 22.0 software (SPSS Inc., Chicago, IL, United States) was used to analyze the data. Before assessing the differences between groups, the Shapiro–Wilk test was firstly used to confirm whether the variables exhibited a normal distribution. The variables that showed a normal distribution were analyzed by one-way analysis of variance (ANOVA) with a Duncan’s multiple range tests. The variables that showed a non-normal distribution (some data of microbiota taxa diversity) were analyzed by a Kruskal–Wallis test. Additionally, the linear and quadratic trend was performed using orthogonal polynomial contrasts to determine the effects of increasing levels of ESBM, and the statistical difference was significant if *p* < 0.05 and trended if 0.05 ≤ *p* < 0.10.

## 3 Results

### 3.1 Growth performance and economic analysis

**Figure 1:**
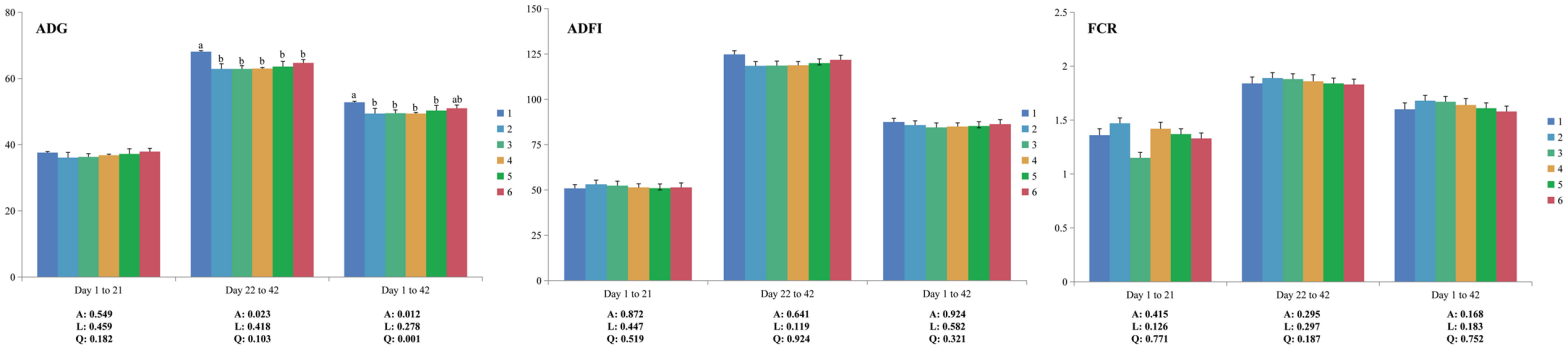
Effects of ESBM supplementation on the growth performance of broiler chickens fed low-CP diets. The values are expressed as the mean values (*n* = 6). Numbers 1 to 6 are the PC, NC, NC + 0.5, 1.0, 1.5, and 2.0% ESBM groups, respectively. A, *p* value of one-way ANOVA; L, *p* value of linear analysis; Q, *p* value of quadratic analysis; ADG, average daily gain; ADFI, average daily feed intake; FCR, feed conversion ratio. ^a,b^Mean values with different letters were significantly different (*p* < 0.05).

No mortality was observed throughout the experiment. From 1 to 21 days of age, there were no significant differences (*p* > 0.05) in ADG, ADFI and FCR among all groups (Figure 1). Compared to PC, NC showed decreased (*p* < 0.05) ADG at both the grower period (22–42 days of age) and whole period (1–42 days of age) but had no significant differences (*p* > 0.05) in ADFI and FCR. Increasing dietary levels of ESBM did not influence (*p* > 0.05) ADG, ADFI and FCR in broilers fed low-CP diets. Notably, during 1–42 days of age, the level of ESBM was quadratically correlated (*p* < 0.05) with ADG in broilers, and the ADG of the 2.0% ESBM group was comparable with that of the PC group.

Results from Table 3 showed that compared to PC, NC had decreased (*p* < 0.05) feed cost per kg, total feed cost per bird, body weight, body weight gain, total cost, total revenue, net profit and economic efficiency. Dietary supplementation with ESBM had an increased effect (*p* < 0.05) on all economic indicators (including feed cost per kg, total feed cost per bird, body weight, body weight gain, total cost, total revenue, net profit and economic efficiency) except from feed cost per kg weight gain (*p* > 0.05) and showed a linear trend (*p* < 0.05). Compared to the other groups, the 2.0% ESBM group showed comparable (*p* > 0.05) feed cost per kg and total feed cost per bird with PC. Although there was no significant difference (*p* > 0.05) in net profit between the PC and 2.0% ESBM groups, the 2.0% ESBM group had a lower (*p* < 0.05) economic efficiency than the PC group, and the NC group showed the lowest economic efficiency (*p* < 0.05) than other groups.

**Table 3:** Economic analysis of dietary ESBM supplementation in broiler chickens fed low-CP diets.

Items	PC	NC	Added ESBM, %				SEM	*p*-value		
			NC + 0.5%	NC + 1.0%	NC + 1.5%	NC + 2.0%		A	L	Q
Feed cost/kg, ¥ (including ESBM)	4.34^a^	4.27^e^	4.28^d^	4.30^c^	4.32^b^	4.33^a^	0.03	<0.01	<0.01	0.875
Total feed cost/bird, ¥	15.99^a^	15.28^b,c^	15.18^c^	15.38^b,c^	15.55^b,c^	15.92^a^	0.47	0.002	0.003	0.140
Body weight, kg	2.24^a^	2.12^b^	2.12^b^	2.15^a,b^	2.17^a,b^	2.22^a,b^	0.07	<0.01	<0.01	0.302
Body weight gain, kg	2.20^a^	2.08^b^	2.08^b^	2.11^a,b^	2.13^a,b^	2.18^a,b^	0.08	0.008	0.003	0.210
Feed cost/kg weight gain, ¥	7.38	7.41	7.41	7.40	7.32	7.34	0.22	0.998	0.806	0.552
Total cost, ¥	21.29^a^	20.55^b,c^	20.48^c^	20.68^a,b,c^	20.85^a,b,c^	21.22^a,b^	0.47	0.018	0.006	0.208
Total revenue, ¥	21.95^a^	20.77^b^	20.76^b^	21.08^a,b^	21.29^a,b^	21.75^a,b^	0.71	0.004	0.001	0.297
Net profit/kg weight gain, ¥	0.30^a^	0.11^e^	0.13^d,e^	0.17^c,d^	0.21^b,c^	0.26^a,b^	0.08	<0.01	<0.01	0.343
Economic efficiency, %	4.11^a^	1.43^f^	1.78^e^	2.63^d^	2.82^c^	3.37^b^	0.96	<0.01	<0.01	0.065

Broiler chickens were fed a standard high-CP diet (PC), a low-CP diet (NC), or an NC diet supplemented with 0.5, 1.0, 1.5%, or 2.0% ESBM; A, *p* value of one-way ANOVA; L, *p* value of linear analysis; Q, *p* value of quadratic analysis. Fixed costs of all groups = 5.3 ¥ and selling prices of per kg live broiler weight = 9.8 ¥. Total cost (TC) = total feed cost + fixed cost. Total revenue (TR) = body weight × selling price. Net profit (NP) = TR – TC. Economic efficiency (EE) = NP/feed cost per kg weight gain × 100. ^a,b,c,d,e,f^Mean values within a row with different superscript letters were significantly different (*p* < 0.05).

### 3.2 Organ indices

As shown in Table 4, at 21 days of age, significant differences (*p* < 0.05) in proventriculus and gizzard indices were observed in both the PC and NC groups. Compared to NC, the addition of ESBM to the diet had an effect (*p* < 0.05) on the pancreas, proventriculus and gizzard indices, and both linear and quadratic effects were also observed in the pancreas and gizzard indices (*p* < 0.05). Compared to the other groups, the 2.0% ESBM group showed the highest pancreas, proventriculus and gizzard index values.

**Table 4:** Effects of ESBM supplementation on the organ index of broiler chickens fed low-CP diets (g/kg).

Items	PC	NC	Added ESBM, %				SEM	*P*-value		
			NC + 0.5%	NC + 1.0%	NC + 1.5%	NC + 2.0%		A	L	Q
**Day 21**										
Liver	23.00	21.99	21.79	23.57	22.30	23.10	0.70	0.116	0.093	0.580
Pancreas	3.14^b,c^	2.96^c,d^	3.05^c,d^	3.10^b,c^	3.29^a,b^	3.41^a^	0.08	0.001	0.041	0.001
Proventriculus	4.81^b^	5.50^a^	5.38^a^	5.04^a,b^	5.26^a,b^	5.55^a^	0.25	0.031	0.962	0.050
Gizzard	17.65^c^	19.13^a,b^	18.27^b,c^	17.86^b,c^	19.82^a^	20.00^a^	0.62	0.002	0.025	0.012
**Day 42**										
Liver	18.46^b,c^	17.37^d^	18.00^c^	18.92^a,b^	19.12^a^	19.28^a^	0.26	0.001	0.001	0.027
Pancreas	2.38^b^	2.39^b^	2.39^b^	2.58^a,b^	2.74^a^	2.77^a^	0.10	0.001	0.001	0.959
Proventriculus	3.56^a,b^	3.14^c^	3.47^b^	3.72^a^	3.74^a^	3.74^a^	0.10	0.001	0.001	0.003
Gizzard	15.04^c^	14.58^d^	15.24^c^	16.03^b^	16.00^b^	16.92^a^	0.23	0.001	0.001	0.628

Broiler chickens were fed a standard high-CP diet (PC), a low-CP diet (NC), or an NC diet supplemented with 0.5, 1.0, 1.5%, or 2.0% ESBM; A, *p* value of one-way ANOVA; L, *p* value of linear analysis; Q, *p* value of quadratic analysis. Organ index = organ weight (g)/live body weight (kg). ^a,b,c,d^Mean values within a row with different superscript letters were significantly different (*p* < 0.05).

At 42 days of age, there were significant differences (*p* < 0.05) in liver, proventriculus and gizzard indices between the PC and NC groups. Dietary supplementation with ESBM significantly influenced (*p* < 0.05) indices of the liver, pancreas, proventriculus and gizzard, in which linear effects (*p* < 0.05) were observed with increasing dietary levels of ESBM. In addition, quadratic effects (*p* < 0.05) of ESBM levels on liver and proventriculus indices were also observed. Among all groups, the 2.0% ESBM group had the highest liver, pancreas, gizzard and proventriculus indices.

### 3.3 Cecal VFAs

As shown in Table 5, the PC group had a higher acetic acid concentration at 21 days of age and propionic acid concentration at both 21 and 42 days of age than the NC group (*p* < 0.05). At 21 days of age, dietary supplementation with ESBM significantly affected (*p* < 0.05) acetic acid, propionic acid and butyric acid concentrations in the cecum in a linear manner. At 42 days of age, the cecal propionic acid concentration was significantly influenced (*p* < 0.05) by dietary supplementation with ESBM but not acetic acid and butyric acid (*p* > 0.05). Increasing dietary ESBM levels linearly increased (*p* < 0.05) the cecal propionic acid concentration at 42 days of age.

**Table 5:** Effects of ESBM supplementation on cecal VFA concentrations of broiler chickens fed low-CP diets (mmol/L).

Items	PC	NC	Added ESBM, %				SEM	*p*-value		
			NC + 0.5%	NC + 1.0%	NC + 1.5%	NC + 2.0%		A	L	Q
**Day 21**										
Acetic acid	15.36^a^	12.35^b^	15.94^a^	15.96^a^	15.39^a^	14.93^a^	0.53	0.001	0.001	0.001
Propionic acid	0.59^a^	0.53^b^	0.56^a^	0.60^a^	0.59^a^	0.60^a^	0.02	0.002	0.001	0.073
Butyric acid	3.81^b,c^	3.70^c^	4.05^a,b^	4.07^a,b^	4.13^a,b^	4.22^a^	0.14	0.024	0.001	0.188
**Day 42**										
Acetic acid	13.59	12.22	13.29	14.21	14.52	14.16	1.11	0.320	0.049	0.249
Propionic acid	1.28^a^	1.08^b^	1.21^a,b^	1.29^a^	1.38^a^	1.31^a^	0.08	0.028	0.001	0.074
Butyric acid	3.51	3.48	3.80	3.78	3.75	3.79	0.14	0.062	0.076	0.137

Broiler chickens were fed a standard high-CP diet (PC), a low-CP diet (NC), or an NC diet supplemented with 0.5, 1.0, 1.5%, or 2.0% ESBM; A, *p* value of one-way ANOVA; L, *p* value of linear analysis; Q, *p* value of quadratic analysis. ^a,b,c^Mean values within a row with different superscript letters were significantly different (*p* < 0.05).

### 3.4 Cecal microbiota

**Figure 2:**
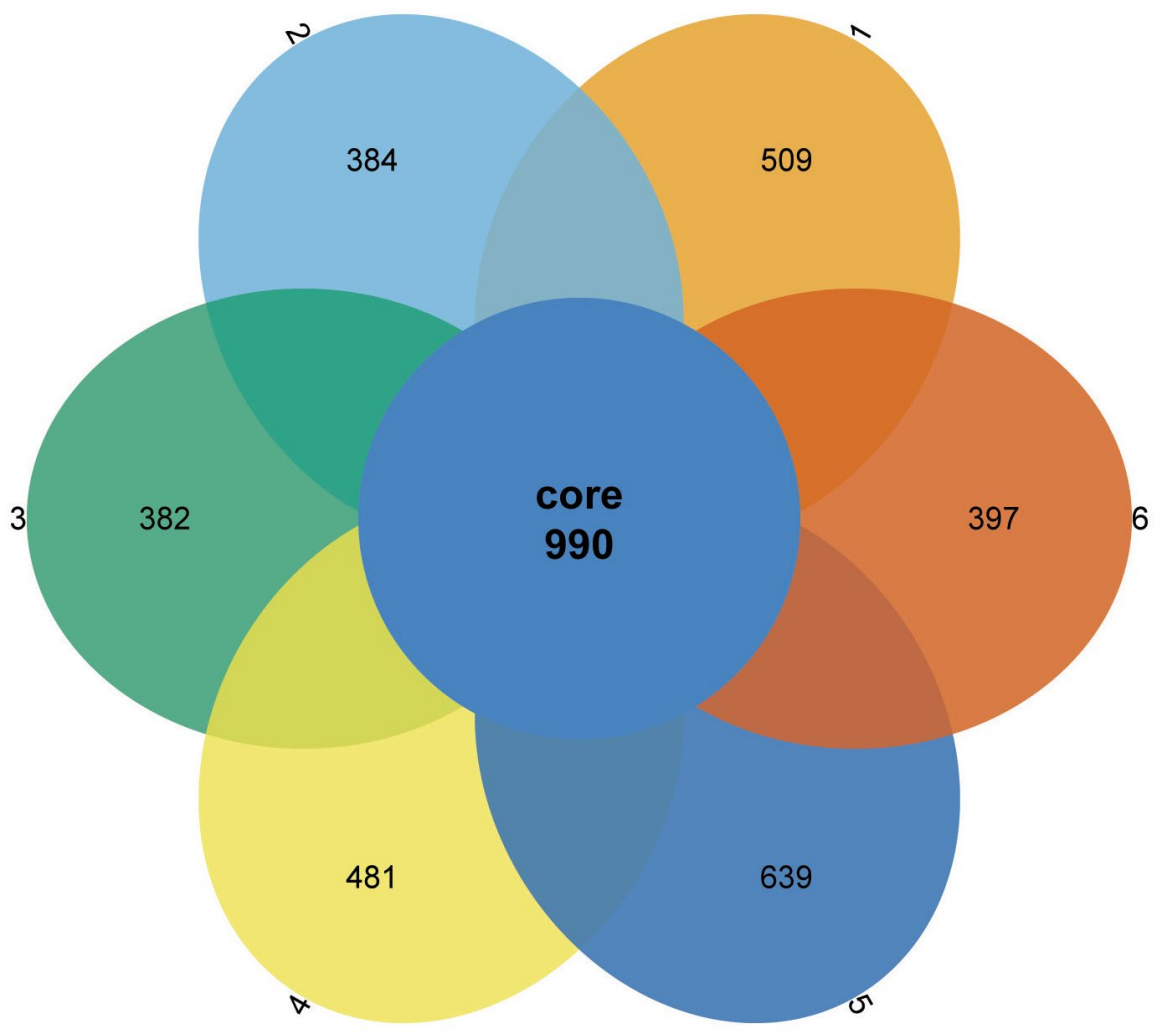
Venn diagram showing the shared and unique operational taxonomic units (OTUs) in the cecal digesta of broilers at 42 days of age. The values were expressed as the mean values (*n* = 6). Numbers 1–6 are the PC, NC, NC + 0.5, 1.0, 1.5, and 2.0% ESBM groups, respectively.

**Figure 3:**
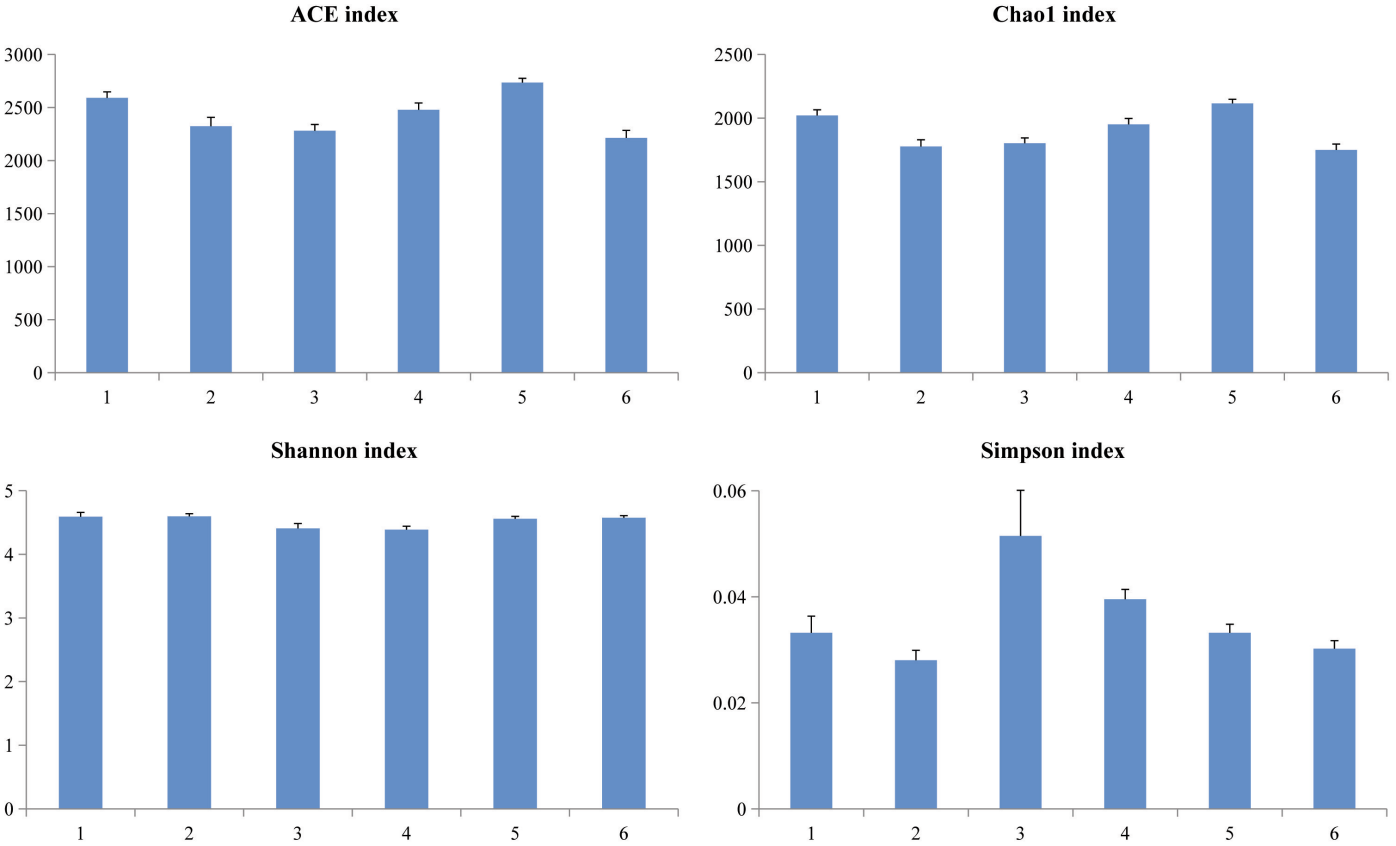
Richness and diversity of the microbial community estimated by Chao1, ACE, Shannon and Simpson in the cecal digesta of broilers at 42 days of age. The values were expressed as the mean values (*n* = 6). Numbers 1–6 are the PC, NC, NC + 0.5, 1.0, 1.5, and 2.0% ESBM groups, respectively.

A Venn diagram was used to compare the similarities and differences between the microbial communities of the different groups. As shown in Figure 2, 990 core OTUs were found to be distributed in all groups. Compared to the PC group, the NC group showed decreased specific OTUs, while the 1.5% ESBM group had a comparable specific OTUs with the PC group. However, there was no significant difference in total OTUs among groups (*p* > 0.05). The alpha diversity analysis was performed for the observation of ACE, Chao1, Shannon and Simpson indices, which were representative of the community richness and diversity. No significant differences in these alpha diversity indices were observed among groups (*p* > 0.05; Figure 3).

**Figure 4:**
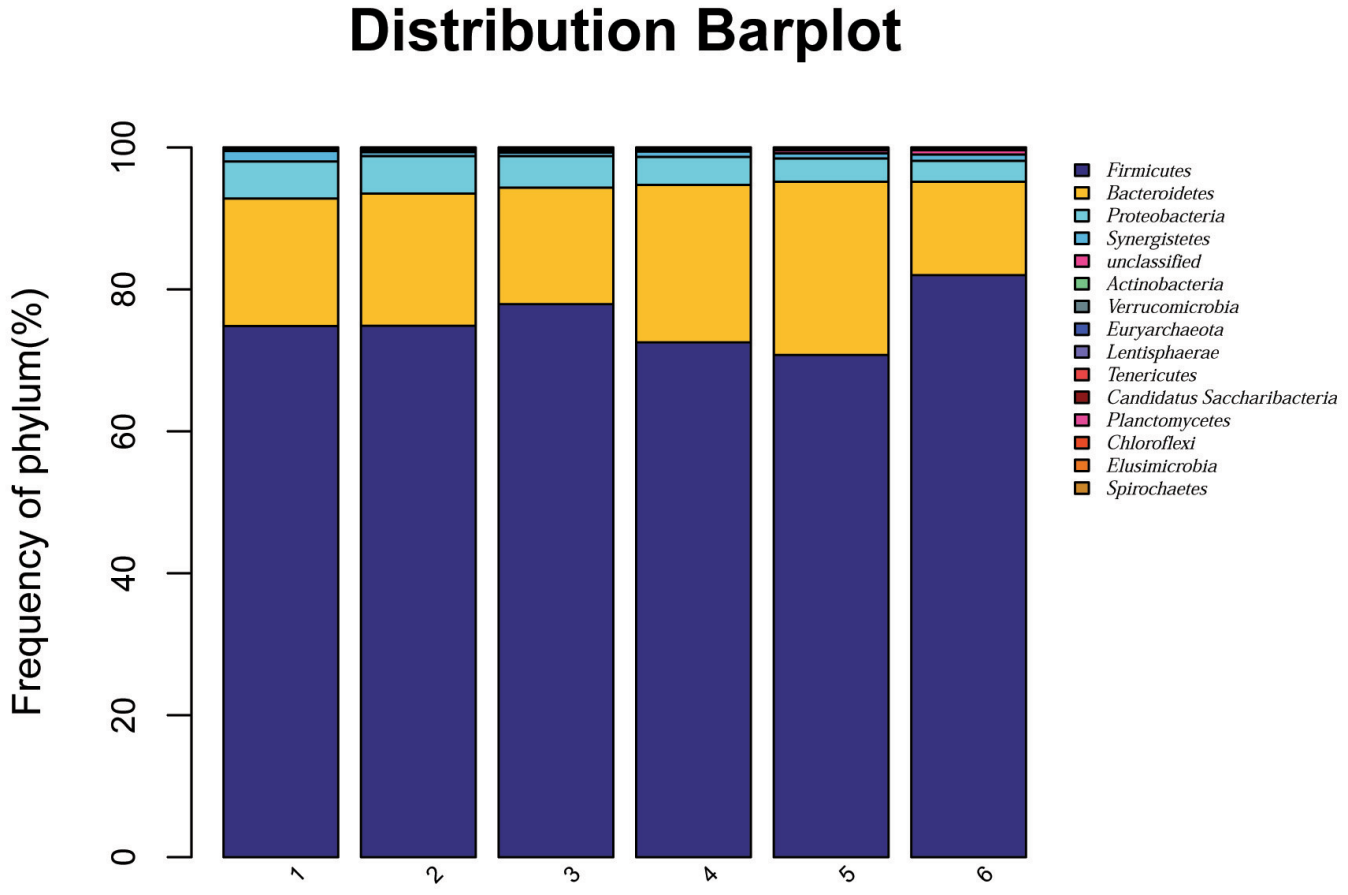
Relative abundance of bacterial phyla in the cecal digesta of broilers at 42 days of age. The values were expressed as the mean values (*n* = 6). Numbers 1–6 are the PC, NC, NC + 0.5, 1.0, 1.5, and 2.0% ESBM groups, respectively.

**Figure 5:**
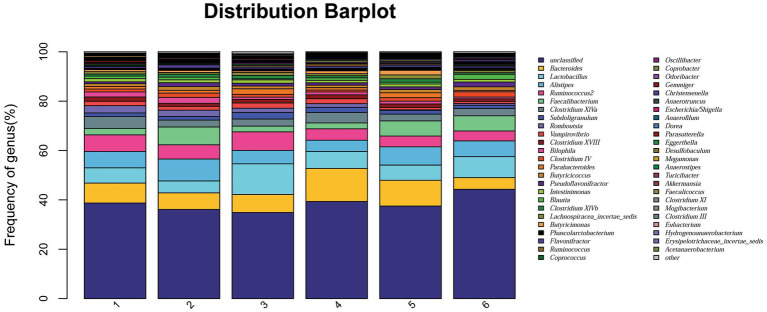
Relative abundance of bacterial genera in the cecal digesta of broilers at 42 days of age. The values were expressed as the mean values (*n* = 6). Numbers 1–6 are the PC, NC, NC + 0.5, 1.0, 1.5, and 2.0% ESBM groups, respectively.

At the phylum level, a total of 15 phyla were identified from 36 cecal samples (Figure 4). Among those, 8 phyla with relative abundances >0.01% were present and Firmicutes, Bacteroidetes, Proteobacteria and Synergistetes were more predominant than others. No significant differences (*p* > 0.05) were found among groups, although the 2.0% ESBM group showed a higher abundance of Firmicutes and a lower abundance of Bacteroidetes and Proteobacteria than the other groups (Table 6). At the genus level, 50 genera with abundance >0.1% were detected (Figure 5). Among those, 18 genera had an abundance above 1% in all groups. There was no significant difference (*p* > 0.05) in the abundance of genera between the PC and NC groups. However, with increasing levels of ESBM, the abundance of *Bacteroides*, *Faecalibacterium* and *Clostridium IV* quadratically increased (*p* < 0.05), and those of *Ruminococcus*, *Romboutsia*and *Blautia* linearly decreased (*p* < 0.05; Table 7).

**Table 6:** Effects of ESBM supplementation on relative abundance of cecal bacteria at the phylum level in broiler chickens fed low-CP diets (%).

Items	PC	NC	Added ESBM, %				SEM	*p*-value		
			NC + 0.5%	NC + 1.0%	NC + 1.5%	NC + 2.0%		A	L	Q
Firmicutes	74.71	75.66	78.3	72.09	71.12	82.03	3.34	0.231	0.602	0.092
Bacteroidetes	18.41	17.82	16.12	22.76	23.90	12.83	4.29	0.475	0.872	0.141
Proteobacteria	4.90	5.29	4.38	3.85	3.34	3.15	1.24	0.788	0.183	0.751
Synergistetes	1.49	0.58	0.51	0.72	0.79	0.94	0.26	0.128	0.228	0.764

Broiler chickens were fed a standard high-CP diet (PC), a low-CP diet (NC), or an NC diet supplemented with 0.5, 1.0, 1.5%, or 2.0% ESBM (*n* = 6); A, *p* value of one-way ANOVA; L, *p* value of linear analysis; Q, *p* value of quadratic analysis.

**Table 7:** Effects of ESBM supplementation on relative abundance of cecal bacteria at the genus level in broiler chickens fed low-CP diets (%).

Items	PC	NC	Added ESBM, %				SEM	*p*-value		
			NC + 0.5%	NC + 1.0%	NC + 1.5%	NC + 2.0%		A	L	Q
*Bacteroides*	8.14	6.45	7.21	13.70	10.33	4.44	2.52	0.173	0.110	0.020
*Faecalibacterium*	2.76	6.95	2.31	2.31	6.23	6.53	1.56	0.089	0.537	0.024
*Clostridium IV*	1.25	1.76	1.13	0.79	1.39	1.94	0.40	0.378	0.625	0.035
*Ruminococcus*	6.66	5.58	7.56	4.57	4.27	3.99	0.92	0.059	0.034	0.597
*Romboutsia*	2.83^a^	2.77^a^	1.85^a,b^	1.56^a,b^	0.71^b^	0.20^b^	0.56	0.011	0.003	0.401
*Blsutia*	1.05^a^	1.12^a^	1.06^a^	0.98^a,b^	0.76^a,b^	0.45^b^	0.27	0.006	0.015	0.004

Broiler chickens were fed a standard high-CP diet (PC), a low-CP diet (NC), or an NC diet supplemented with 0.5, 1.0, 1.5%, or 2.0% ESBM (*n* = 6); A, *p* value of one-way ANOVA; L, *p* value of linear analysis; Q, *p* value of quadratic analysis. ^a,b^Mean values within a row with different superscript letters were significantly different (*p* < 0.05).

## 4 Discussion

The present findings indicated that supplementation with ESBM had no compromising effect on the growth performance in broilers fed low-CP diets. Feeding a low-CP diet to livestock and poultry is conducive to reducing the cost of animal breeding and alleviating nitrogen emissions in animal production. However, an excessive decrease in the dietary CP level could lead to weakened feed utilization associated with an increase in fat deposition (19, 20). In addition, the inclusion of more nonbound amino acids into a low-CP diet may increase dietary starch:protein ratios, leading to an imbalanced digestion dynamic between energy and nitrogen, which has negative consequences for nutrient digestion, growth performance and intestinal health in broilers (21). The current results suggested that reducing the CP level in the diet at 10 g/kg decreased ADG from 22 to 42 days and from 1 to 42 days. Similar to our results, Hejdysz et al. (22) also found that feeding broilers low-CP diets had no negative effect on growth performance in the starter period, while ADG was decreased in the grower, finisher and whole periods when the dietary CP level was reduced by 1 to 3%. Although free amino acids were added to a low-CP diet to meet nitrogen requirements, an imbalance between energy and protein may hamper nutrient digestion and absorption, thus leading to compromised performance. Compared to other protein feedstuffs, such as SBM, ESBM has reduced amounts of antinutritional factors and increased amounts of peptides and AAs, thus facilitating intestinal solubility absorption (15). Yu et al. (16) reported that ESBM could replace up to 75% of fish meal in the diet without a significant effect on the growth performance of abalone. The results from Li et al. (23) showed that piglets fed ESBM had comparable ADG, ADFI and FCR to those fed fish meal and milk powder. In this study, we found that ESBM supplementation at a level of 2.0% in low-CP diets could maintain ADG and even attain ADG similar to that of broilers fed a standard high-CP diet from 1 to 42 days, suggesting that ESBM could be an effective substitute for SBM to be used in low-CP diets.

Broiler production is an important portion of poultry industry worldwide which is driven by economic feasibility and optimization, and one of the key challenges for broiler producers is to increasing broilers’ productivity while remaining economically sustainable (24). Lowering the dietary CP level is desired and can be of critical economic consideration as protein and amino acids are costly nutrients per unit feed weight (5). In this study, reducing CP level by 1% in the diet indeed decreased the cost of feed compared to the standard high CP diet. However, due to a lower body weight and body weight gain, a total revenue was significantly decreased in broilers fed a low-CP diet than those fed a standard high-CP diet, thus leading to a low net profit and economic efficiency per unit body weight gain. Because the price of ESBM (8.0 ¥/kg) was higher than that of SBM (5.0 ¥/kg) at the time of the experiment, increasing levels of ESBM in a low-CP diet increased the feed cost in this study. Notably, both numerically increased body weight and body weight gain compensated for the total cost, maintained the total revenue and increased net profit and economic efficiency in broilers fed increasing ESBM in a low-CP diet. Results from this study also showed that the body weight of broilers at 42 days of age was relatively lower than the current dominating market weight, thus narrowing the net profit and lowering the economic efficiency. The reason may be that broilers in this study were fed diets in the form of mash, not crumble or pellet, leading to lower feed intake and body weight. Massuquetto et al. (25) and Brink et al. (7) have demonstrated that broilers fed a pellet diet had increased feed intake and body weight than those fed a mash diet at market age. In addition, the rearing system for broilers was also a consideration. In China, broiler producers prefer a cage-rearing system instead of a floor-rearing system due to its higher economic benefits (26). Therefore, the application prospect and economic benefit of ESBM in broilers reared in different systems should be studied further.

Digestive organ indices can reflect the status of development and function of digestion in animals. The proventriculus is an organ that has many glands to secrete hydrochloric acid and pepsin, and the gizzard performs functions by mechanically grounding feedstuffs into small particles and mixing these particles with digestive fluids, thus contributing to nutrient digestion. The pancreas secretes fluids that contain trypsinogen, lipase and amylase, and is an important digestion assistant organ. The liver is a site where glucose, fatty acid and amino acid metabolism occurs, playing a central pivotal role in nutrient metabolism (27). Jariyahatthakij et al. (28) reported that reducing the dietary CP level from 21 to 18% increased the liver weight of broilers at 24 days of age. The results from Allameh and Toghyani (19) and Chrystal et al. (29) showed that the dietary CP level had no effects on the pancreas and gizzard weights of broilers at 35 days of age. In this study, our results suggested that broilers fed a low-CP diet had increased relative weights of the proventriculus and gizzard at 21 days of age but decreased weights of the proventriculus, gizzard and liver at 42 days of age, indicating that the CP level in the diet can affect the development of these digestive organs. Presumably, the proventriculus and gizzard are the main sites of nutrient digestion, and the liver is the site of nutrient metabolism, both of which are more sensitive to nutrient disturbance, such as a change in CP level (22). Inclusion of ESBM in a low-CP diet increased the relative weights of the pancreas and gizzard at 21 days of age and that of the liver, pancreas, proventriculus and gizzard at 42 days of age in this study, and the 2% ESBM group showed the highest value, suggesting that a low-CP diet supplemented with ESBM could contribute to the development of digestive organs in broilers. A reduction in dietary CP was reported to decrease the weights of digestive organs, such as gizzard and pancreas (30), and the development of digestive organs were significantly associated with feed efficiency, in which a better feed efficiency led to an increased body weight and positively correlated with the relative weights of digestive organs in broilers (31). In this study, ESBM supplementation in the low-CP diets could enhance the ADG and feed efficiency, which may positively affect the development of digestive organs in broilers. To date, little information on the effect of ESBM on the development of organs has been attained, and the mechanism underlying this effect should be studied further.

Dietary undigested substrates including nonstarch polysaccharides, starch and amino acids, can be fermented by intestinal microorganisms into VFAs, which have been demonstrated to have beneficial effects on energy metabolism, intestinal health and immune responses in broilers (32). Little information on the effects of a low-CP diet on VFA production in broilers has been acquired. In this study, we found that broilers fed a low-CP diet had decreased concentrations of acetic acid and propionic acid compared with those fed a standard high-CP diet. Presumably, replacing vegetable protein feed with crystalline free amino acids in a low-CP diet may decrease the fermentation substrates, such as cell wall polysaccharides, by intestinal microorganisms into VFAs. Compared to conventional SEM, ESBM was reported to have reduced levels of stachyose and raffinose, which can be fermented into VFAs by intestinal microorganisms (33). However, our results showed that an increased level of acetic acid and propionic acid in the cecal digesta was associated with an increased inclusion of ESBM in a low-CP diet. Results from Ruckman et al. (34) showed a linear increase in acetate, propionate, butyrate, and total VFAs concentration in ileal digesta associated with an increase in the inclusion level of ESBM in piglets, and the reason may be that the inclusion of ESBM may slow the digesta transit rate, giving microbes in the gut more time to ferment substrates, such as nonstarch polysaccharides, to produce VFAs. Compared to SBM, ESBM had a more digestible protein content, which was demonstrated that an increase in digestible dietary protein positively affected total cecal VFAs concentration in broilers (35). In this study, adding ESBM into a low-CP diet may stimulate specific bacteria growth and enhance microbial activity by optimizing the balance between the energy and nitrogen in the gut, thus contributing to fermentation and producing more VFAs.

The importance of the gut microbiota is widely acknowledged because of its important roles in improving the health and performance of broilers (36). Diet has a great influence on regulating the gut microbiota and the metabolites of the host. Bacteria belonging to the Firmicutes, Bacteroides, and Proteobacteria phyla constitute the dominant microbiota in broilers (37). The results from the current study showed that the most abundant phyla identified in the cecal contents were Firmicutes, Bacteroidetes and Proteobacteria in broilers at 42 days of age. This is in accordance with previous studies showing that Firmicutes, Bacteroidetes and Proteobacteria are the most dominant phyla in the cecum of 42-day-old broilers (36). The CP level in the diet is an important factor in regulating the gut bacterial community. However, in this study, we found that lowering the CP level of the diet by 1% did not alter the cecal microbial composition of broilers at 42 days of age. An explanation may be that adding crystalline free amino acids into a low-CP diet can meet the demand for nitrogen by the gut microbiota and maintain the microbial activity, thus leading to a stable microbial structure. The results from this study showed that the abundances of *Bacteroides*, *Faecalibacterium* and *Clostridium IV* were increased in the ESBM group. *Bacteroides* were found to possess a fascinating array of enzymes that can degrade complex dietary substrates into VFAs (38). Huang et al. (39) reported that *Bacteroides* was a potential biomarker of high feed efficiency, in which the cecal abundance of *Bacteroides* was negatively correlated with FCR values in broilers. A study from Stappenbeck et al. (40) revealed that during the development of posterior angiogenesis, *Bacteroides* stimulated angiogenesis, thus contributing to the formation of the capillary network for the efficient distribution of absorbed nutrients in mice, implying that *Bacteroides* could act as environmental agents that shape development of the intestinal villus microvasculature and increase the intestine’s absorptive capacity, providing benefit to the host. Although there was no difference in FCR among groups, an increased abundance of *Bacteroides* may have an influence on host nutrient absorption, thus resulting in an increased ADG in the ESBM groups. *Faecalibacterium*is one of the most abundant symbiotic bacteria in the intestine of broilers. It can protect intestinal health by adhering to the intestinal tract through the exclusion effect to form the intestinal barrier (41). Zhou et al. (42) reported that *Faecalibacterium* was often related to healthy conditions and that its reduction usually occurred under diseased conditions. In addition, *Clostridium IV*, including *C. leptum*, *C. sporosphaeroides*, *C. cellulosi* and *Faecalibacterium prausnitzii*, was reported to break complex carbohydrates down into VFAs in the gut, which were beneficial to intestinal epithelial cells (43). Our results in this study indicated that dietary supplementation with ESBM could increase the cecal abundances of *Faecalibacterium* and beneficial *Clostridium IV*, which will be conducive to the health of broilers.

In conclusion, the results from the current study indicate that decreasing the CP level by 1% in the diet had a negative effect on growth performance, economic efficiency, organ development and cecal fermentation in broilers. Supplementation with ESBM at a level of 2.0% in a low-CP diet could improve ADG, economic efficiency and digestive organ indices associated with cecal fermentation and intestinal microbiota in broilers. Thus, ESBM may be a potential soybean protein feed to improve growth and health by modulating the microbiota in broilers offered a low-CP diet.

## Data availability statement

The original contributions presented in the study are publicly available. This data can be found here: https://www.ncbi.nlm.nih.gov/bioproject; PRJNA1019461.

## Ethics statement

The animal study was approved by Animal Care and Use Committee of Shenyang Agricultural University. The study was conducted in accordance with the local legislation and institutional requirements.

## Author contributions

XZ: Writing – original draft. KG: Investigation, Writing – original draft. YQ: Data curation, Writing – original draft. GY: Supervision, Writing – review & editing. HL: Conceptualization, Writing – review & editing.
