# Supplementary material for: Enzymolytic soybean meal improves growth performance, economic efficiency and organ development associated with cecal fermentation and microbiota in broilers offered low crude protein diets
Source: Front Vet Sci. 2023 Nov 17;10:1293314. doi: 10.3389/fvets.2023.1293314 (PMC10693456; doi:10.3389/fvets.2023.1293314)
Supplement: Supplementary file 1 [file Table_1.docx]

Supplementary Material

**Table S1** Nutrition and anti-nutrition factor level and amino acid composition of SBM and ESBM (air-dried basis, %)

| Items | SBM | ESBM | Items | SBM | ESBM |
| --- | --- | --- | --- | --- | --- |
| Nutrient level, % | | | | | |
| Dry matter | 86.98 | 89.82 | Crude ash | 6.21 | 6.14 |
| Crude protein | 45.14 | 48.26 | TCA-SP | 1.87 | 18.98 |
| Amino acid composition, % | | | | | |
| Indispensable amino acids |  |  | Dispensable amino acids |  |  |
| Arginine | 3.38 | 3.52 | Alanine | 1.48 | 1.58 |
| Histidine | 1.10 | 1.09 | Aspartic acid | 5.41 | 5.81 |
| Isoleucine | 1.75 | 2.16 | Cystine | 1.42 | 1.46 |
| Leucine | 3.86 | 4.32 | Glutamic acid | 8.77 | 9.46 |
| Lysine | 2.75 | 2.73 | Serine | 2.42 | 2.48 |
| Methionine | 0.47 | 0.44 | Tyrosine | 1.11 | 1.15 |
| Phenylalanine | 2.33 | 2.59 | Glycine | 1.82 | 1.90 |
| Threonine | 1.72 | 1.88 | Proline | 2.68 | 2.66 |
| Tryptophan | 0.55 | 0.67 |  |  |  |
| Valine | 1.70 | 2.06 |  |  |  |
| Anti-nutrition factor, mg/g | | | | | |
| Glycinin | 128.95 | 2.58 | β-conglycinin | 95.14 | 4.91 |
| Trypsin inhibitor | 9.69 | Not detected |  |  |  |

SBM, soybean meal; ESBM, enzymolytic soybean meal; TCA-SP, trichloroacetic acid soluble protein.
